# The coordination of unprotonated peptide tertiary structure as a metric of pMHC–TCR functional avidity

**DOI:** 10.1016/j.dib.2015.09.009

**Published:** 2015-09-28

**Authors:** Georgios S.E. Antipas, Anastasios E. Germenis

**Affiliations:** a Division of Materials Technology, National Technical University of Athens, Zografou Campus, Athens 15780, Greece; b Department of Immunology & Histocompatibility, School of Medicine, University of Thessaly, Biopolis, Larissa 41110, Greece

**Keywords:** pMHC–TCR interaction, Atomic pair correlation, Short range order, Cumulative coordination, Functional avidity, Structure–function relationship

## Abstract

The coordination difference between the unprotonated tertiary structures of a native (Tax) peptide and a number of its variants – all peptides presented by HLA-A201 and bound to the human A6 T cell receptor-was discovered to constitute a metric of pMHC–TCR functional avidity. Moreover, increasing coordination deviations from the index were found to flag correspondingly weakening immunological outcomes of the variant peptides. The prognostic utility of the coordination difference of unprotonated tertiary structure was established to operate strictly on the peptide scale, seizing to be of relevance either to the immediate peptide environment (i.e. within the realm of peptide short range order, within 7 Å of any peptide atom) or over the entirety of the pMHC–TCR complex. Additionally, the imprint of peptide immunological identity was expressed both by the total coordination as well as by its C–C partial.

**Specifications Table**

**Table:** 

Subject area	*Immunology, Biochemistry, Materials Science, Quantum Chemistry*
More specific subject area	*Class I MHC, CD8+ Cytotoxic Lymphocytes, Protein-protein interactions*
Type of data	*Excel spreadsheet*
How data was acquired	*Data from crystallized tertiary structures was acquired from the Protein Data Bank (PDB)*
Data format	*Text*
Experimental factors	*None*
Experimental features	*None*
Data source location	*Not applicable*
Data accessibility	*Data is with this article*

**Value of the data**

• The current data and underlying methodology may serve as a benchmark towards further investigations of peptide functionality in respect to peptide unprotonated tertiary structure, e.g. from published pMHC–TCR complexes, with emphasis on the behavior of the C–C partial.
• Consistent over- and under-coordination of agonist and antagonist peptides respectively were observed to in comparison to the index. Additionally, coordination selective to the agonizts was found to be reflected on the C–C partial.
• The interatomic distance threshold of 7 °A beyond which coordination appears to be correlated to functional avidity, could be relevant to more pMHC–TCR Class I complexes and the comparison between peptide coordination differences could be portrayed alongside functional avidity differences for further validation of the former as a reliable criterion of peptide biological functionality.

## 1 Data

Our work on the transcriptional regulatory Tax protein of the human T-cell leukemia virus type 1 (HTLV-1) [1–3], indicated that the immunological outcome of a number of functionally diverse variant peptides [4] recognized by the human A6 TCR, was highly correlated to their atomic coordination differences in respect to Tax [5]: over-coordination signified an agonist while under-coordination indicated an antagonist or null peptide. Additionally, gas-phase molecular orbital interactions on protonated tertiary structures revealed that the atomic coordination of agonist peptides resulted in the presence of a stable ammonium group on their N termini which was altogether unattainable for antagonists, and this finding was consistent across the range of conditions studied in regard to peptide formal charge and protonation of side chain groups [5,6]. Interestingly, we also attained data indicating that the atomic coordination of the peptide on the isolated pMHC (in the absence of TCR) may also serve as a metric of functional avidity, as we reported for the case of human cytomegalovirus (HCMV) variants [7].

Thus far, calculations of atomic coordination have relied on protonated peptide structures; protonation itself involves the expensive stage of quantum mechanical relaxation of the H species, while introducing an inevitable bias related to the level of theory employed. Consequently, it is desirable to examine whether the non-protonated peptide tertiary structure carries coordination information which may be correlated to the immunological outcome. Accordingly, the set of data presented here portrays that both total coordination as well as the C-C coordination partial of the peptide may qualify as a primary metric which may be employed to assess the functional avidity of the pMHC–TCR complex.

## 2 Materials and Methods

### 2.1 Peptides

**Fig. 1:**
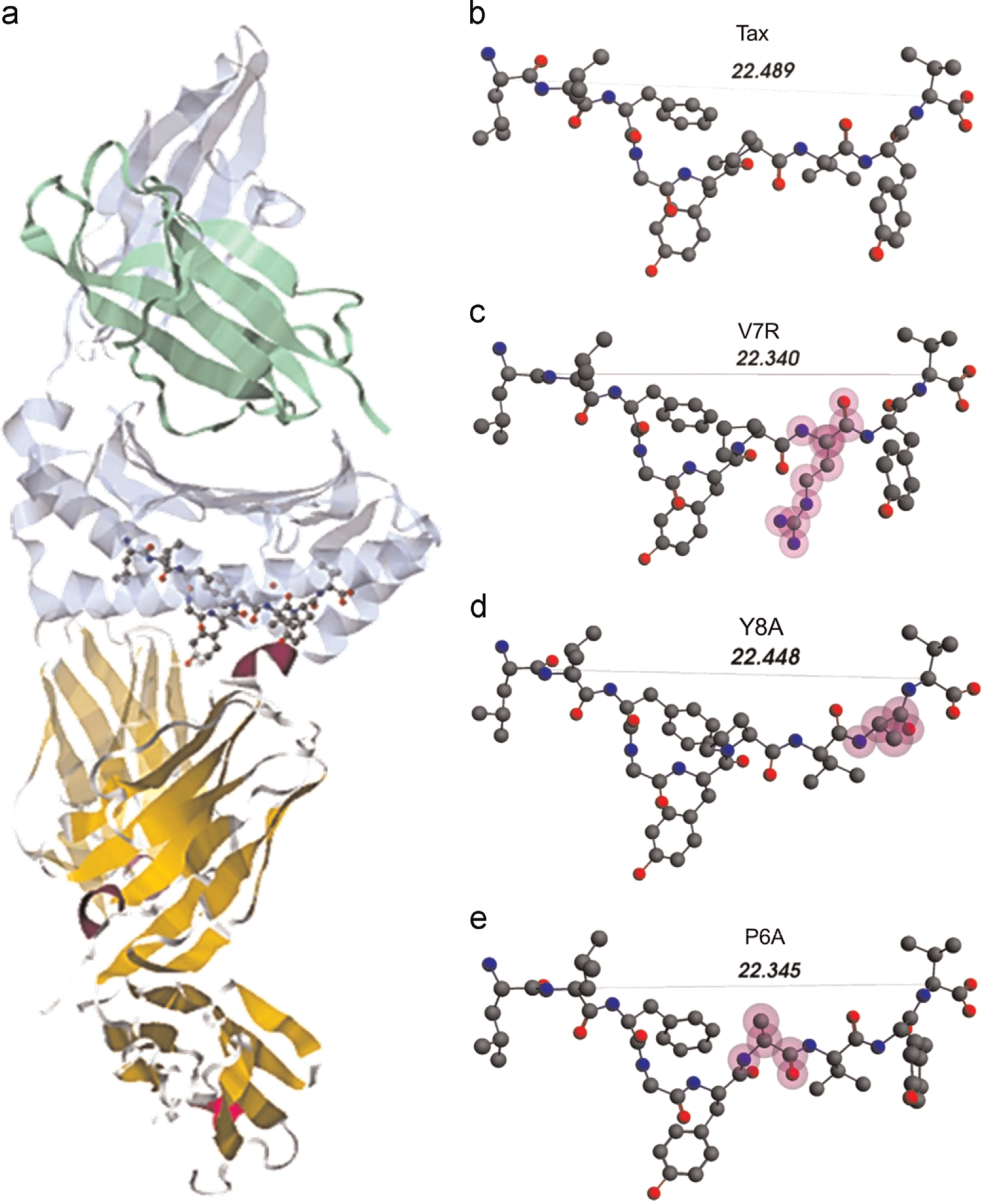
(a) The HLA-A2-Tax-A6 complex. The TCR alpha and beta chains (shown in light yellow and orange color, respectively) are located underneath the peptide, the latter depicted in ball-and-stick format. The MHC alpha chain is located over the peptide (shown as a light cyan ribbon) and encapsulates the peptide’s hydrophobic portion. (b) to (e): unprotonated structures of the Tax, V7R, Y8A and P6A, respectively. In all peptides, hydroxyl groups attached to the phenyl side chain of residue 5 point towards the alpha chain of the TCR. In every structure the distances in °A between the alpha carbon (Cα) atoms of the N- and C-terminus residues are also shown. Atom color notation is C – gray, N – blue and O – red. Peptide mutations in respect to Tax are highlighted in purple.

All peptides were presented by HLA-A201 and were bound to the human A6 TCR [4]. The peptides studied were the cognate HTLV-1 Tax peptide (LLFGYPVYV, PDB entry 1AO7) (index peptide), the weak agonist (or null peptide) V7R (LLFGYPRYV, PDB entry 1QSE), the weak antagonist Y8A (LLFGYPVAV, Protein data bank – PDB entry 1QSF) and the antagonist P6A (LLFGYAVYV, PDB entry 1QRN). The tertiary structures reported here were the peptide chains isolated from the pMHC–TCR complex in each of the PDB complexes. A comparison of these structures is presented in Fig. 1. All pMHC–TCR complexes have been previously characterized by cell assays as well as by kinetic and thermodynamic measurements [4] and on the basis of these measurements, the P6A and Y8A variants are antagonists while Y8A is a weak agonist.

### 2.2 Calculation of pair correlation functions

For all peptide structures, the Pair Distribution Function (PDF) and the Radial Distribution Function (RDF) and were calculated as described in our precursor work [5–7], followed by integration of the RDF which yielded the total and partial atomic coordination. The procedure involved the initial calculation of the histogram of interatomic distances. For every pair of atoms *i* and *j* (*i*≠*j*) on the peptide, their interatomic distance was calculated as

(1) $$r_{ij}=\sqrt{{\left(x_{i}-x_{j}\right)}^{2}+{\left(y_{i}-y_{j}\right)}^{2}+{\left(z_{i}-z_{j}\right)}^{2}}$$

where *x*, *y* and *z* are atomic Cartesian coordinates. Calculation of the peptide Pair Distribution Function (PDF, symbolized as g(r)) is in respect to the real space coordinate, *r*; for the calculation, real space itself is segmented into bins, each bin of a size (width) equal to 0.1 °A Choice of the most appropriate bin size is a matter of experimentation and is particular to the system under investigation [5–7]. On the basis of the interatomic distances, the corresponding histogram, *h*(*r*), may then be constructed as

(2) $$h(r)=\sum_{j=1}^{N-1}\sum_{i>j}^{N}\delta (r-r_{ij})$$

where *N* is the number of peptide atoms and *δ* is the Dirac delta function. The PDF was then calculated as

(3) $$g(r)=\frac{h(r)}{2\pi Nr^{2}\rho_{0}}$$

where *ρ*_0_ is the number density *N*/*V*, *V* is the volume of the simulation box containing the peptide. In (1), the species of the ith and/or jth may be restricted, in which case the *g*(*r*) calculated represents a partial (e.g. if all i atoms are restricted to carbon the PDFs computed would be the carbon partials), otherwise (3) represents the total PDF. The Radial Distribution Function (RDF, symbolized as R(r)), was then calculated as

(4) $$R\left(r\right)=4\pi r^{2}\rho_{0}g\left(r\right)$$

and integrated to estimate the cumulative atomic coordination, *n*^*r*1^, of any atom within a sphere of radius r_1_ as follows

(5) $$n^{r_{1}}=\int_{0}^{r_{1}}R(r)dr=4\pi \rho_{0}\int_{0}^{r_{1}}g(r)r^{2}dr$$

All calculations of PDF, RDF and coordination were performed with the PRDF program [8–11].

**Fig. 2:**
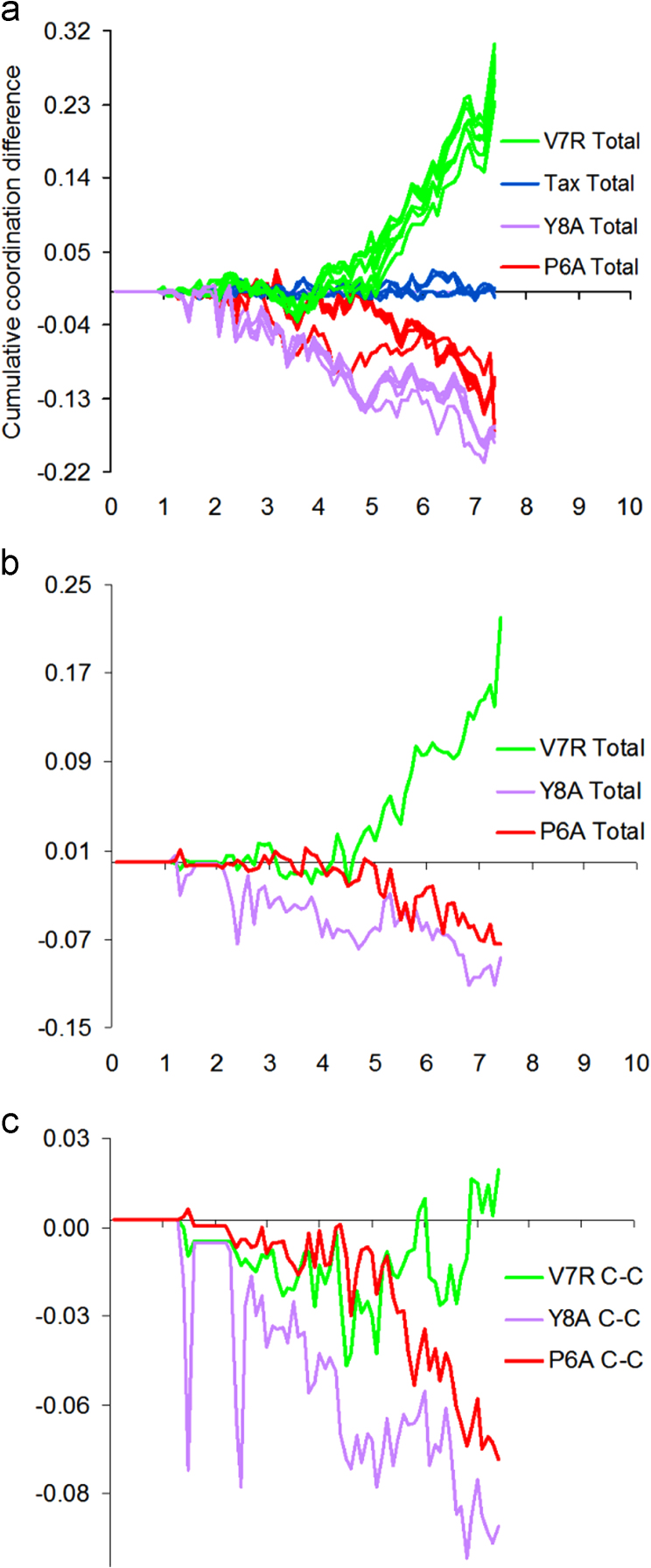
Cumulative coordination differences. (a) Total coordination of protonated peptide tertiary structure models as adapted from the work in [6], (b) total coordination of unprotonated peptide tertiary structures and (c) the C–C partial of unprotonated peptide tertiary structures.

In the current work, PDF motifs of merit (fluctuations) were apparent up to an interatomic distance of approximately 7 °A. Hence, running differences between the coordination of a variant and that of the index were accordingly calculated up to interatomic distances of 7 °A via Eq. (5), for all combinations of partials not involving the H species. These running differences indicated that only the total coordination of unprotonated peptide tertiary structure and its C–C partial (Fig. 2b and c respectively) were correlated to measured functional avidity values of the Tax variants (Fig. 2a).

## 3 Note on the data files

The unprotonated peptide tertiary structures have been included in the file Structures.rar in.xyz format. Structure designation follows from the residue substitution on each peptide, e.g. the file corresponding to the peptide taken from the 1QRN structure is named “P6A.xyz”. Underlying pair correlation data are included in PRDF.rar in comma delimited format. Atomic coordination is compared in PRDF.xls, which comprises total and partial PDF, RDF and RDF(r)dr data in respect to the interatomic distance, *r* (°A). Each of the tabs in PRDF.xls represents a pair correlation partial and lists the underlying PDF (Eq. (3)), RDF (Eq. (4)) and RDF(r)dr (Eq. (5)) data as well as the running sum of RDF(r)dr (coordination) and variant differences in respect to Tax along with graphical representations of these differences.
