# Supplementary material for: The coordination of unprotonated peptide tertiary structure as a metric of pMHC–TCR functional avidity
Source: Data Brief. 2015 Sep 28;5:342–7. doi: 10.1016/j.dib.2015.09.009 (PMC4602356; doi:10.1016/j.dib.2015.09.009)
Supplement: Supplementary file 4 — Supplementary material [file mmc4.docx]

**Conflict of Interest declaration**

07-Sep-2015

The coordination of unprotonated peptide tertiary structure as a metric of pMHC-TCR functional avidity

On behalf of the authors of this paper I declare that there are no conflicts of interest.

Dr Georgios S.E. Antipas

Division of Materials Technology

School of Mining Engineering and Metallurgy

National Technical University of Athens

Zografou Campus

Athens 15780, Greece

Room 3.2B

Tel. +30 210 772 2037

Email: gantipas@metal.ntua.gr

Web: http://users.ntua.gr/gantipas/
